# Group Medical Visits for Addressing Weight and Blood Pressure in an Underserved Population

**DOI:** 10.3390/medicines9120060

**Published:** 2022-12-01

**Authors:** Amit Algotar, Stephanie Trofymenko, Myra Muramoto, Amy Howerter, Randa Kutob

**Affiliations:** College of Medicine, University of Arizona, Tucson, AZ 85724, USA

**Keywords:** blood pressure, obesity, primary healthcare, body weight, community health services

## Abstract

The effect of group medical visits (GMV) compared to individual medical visits (IMV), on weight and blood pressure in a large primary care practice serving a predominantly underserved population, was assessed. The records of 304 patients attending a weight-loss program were analyzed using mixed-effects regression models. Patients in GMV lost an average of 11.63 lbs, whereas patients in IMV lost an average of 3.99 lbs (*p* < 0.001). A total of 55% of patients lost ≥7% in GMV compared to 11% of patients in IMV (*p* ≤ 0.001). Individuals who lost >5% of their baseline weight had a higher reduction in overall blood pressure. For systolic and diastolic blood pressure, the differences between baseline and three months for GMV and IMV were −7.4 vs. 4.1 mm of Hg (*p* = 0.002) and −4.6 vs. 4.2 mm of Hg (*p* = 0.003), respectively. Results from this study demonstrate that GMV may be a potentially useful modality for addressing weight and blood pressure in an underserved population.

## 1. Introduction

The underserved population is disproportionately affected by obesity [1]. Obesity rates among adults without high school education and adults who graduated from college in 2017 were 35.9%, compared to college graduates at 22.7% [2]. In 2017, 47% of Hispanics in the United States were obese, compared to 46.8% of African Americans, 37.9% of Whites, and 12.7% of Asians [2]. Obesity in the United States has a strong inverse relationship with socioeconomic status [3]. There are several barriers to healthcare for underserved and minority groups in the United States. These include financial, structural, and cognitive, which are often interrelated, as described by the Health Care Access Barriers Model (HCAB) [4]. These barriers often result in late presentation and decreased prevention, as well as decreased care, ultimately leading to disparities in health outcomes [4]. It is imperative to develop modalities to reduce these barriers and improve the quality of care that we deliver to our minority patients.

Group medical visits (GMV) are healthcare visits shared alongside similar patients that are conducted by transdisciplinary healthcare teams to increase the time spent with healthcare providers, address physiological and psychological issues, and provide group social support [5]. GMV are a time-efficient and cost-effective modality for delivering quality healthcare [6]. The element of social support has been found to be effective in achieving weight loss in individuals attempting to lose weight [7,8,9]. Studies by Bromely and others have found similar outcomes in applying the GMV approach to weight loss, but few studies are designed to directly compare individual medical visits (IMV) and GMV within the same clinic setting. Many completed studies lack an appropriate control group [10,11,12,13,14]. GMV have reportedly been successful in diabetes care in underserved populations, including veterans, by improving glucose and blood pressure control [14,15,16] as well as improving patient satisfaction [17]. GMV allow for accountability and support that can decrease isolation and improve patient engagement, and are associated with providing an environment for education and support [18,19,20,21]. Most recently, in light of the COVID-19 pandemic, the model of GMV has been successfully adopted to a telehealth platform and implemented virtually [21].

In this paper, we investigated the role of GMV compared to IMV in reducing weight and blood pressure using a retrospective cohort analysis. We hypothesized that when compared to IMV, GMV will demonstrate improved weight loss and blood pressure control.

## 2. Materials and Methods

### 2.1. Population

Data were abstracted from electronic medical records of patients attending the Clinical Weight Loss (CWL) program at Banner University Medical Center—South Campus in Tucson, Arizona, USA, during 2017–2018. The program is housed within a family medicine clinic located in South Tucson, a federally designated medically underserved area where 49.4% of patients treated in the clinic identify as Hispanic or Latino. The median household income is $21,160 with 57.7% households reporting an income less than $25,000 [22].

### 2.2. Program Description

The CWL program is a physician-led medical weight-loss program that offers both GMV and IMV appointment options. The initial visit was a one-on-one medical visit led by a physician consisting of a detailed patient history, full physical examination, and orders for laboratory tests (if indicated). After the initial visit, patients were given the option to schedule their follow-up appointments as GMV or IMV and were scheduled every 2–3 weeks. Patients could schedule the frequency and timing of their visits at their convenience and could participate in the program as long as desired. GMV were conducted by a physician and a health coach. The group included 2–8 patients scheduled during the same 60 min. Patients would rotate one at a time with a private check-in with the physician while the rest of the group met with the health coach. Once all 1:1 check ins were complete, the physician would join the group. In addition to the medical supervision of the physician, patients received lifestyle modification education and support during the group setting. The lifestyle modification was encouraged through increasing self-efficacy using tools such as S.M.A.R.T. (Specific, Measurable, Attainable, Realistic, Timely) goals, nutrition, and physical activity education and general social support as patients discussed their challenges and successes toward achieving their weekly weight-loss goals. Most of the visits were bi-weekly, 60 min in duration, and with a maximum group size of eight patients. IMV were conducted by a physician only (no health coach) in a one-on-one 15 min visit. 

### 2.3. Statistical Analysis

Weight and blood pressure data were collected during each visit conducted every 2–3 weeks. Analyses were restricted to patients who had made three or more visits in the CWL program and weight was included in the visit record. Blood pressure readings, diabetic status, and other diagnoses were extracted if present. *t*-tests were used to describe continuous variables and chi-square tests were used for categorical variables at baseline. Since data were conducted over multiple time points without fixed intervals, mixed-effects regression models were conducted to determine the impact of GMV and IMV on overall weight loss in patients over time. Analyses were adjusted for age, race, and the presence of co-morbidities. Subset analysis was conducted to determine the effect of group medical visits on blood pressure. Analyses was performed using STATA14 statistical software (StataCorp, College Station, TX, USA). 

## 3. Results

The data from 304 patients who attended the clinical weight-loss clinic for this retrospective cohort study are demonstrated in Table 1. Most subjects, n = 198 (65%), participated in IMV, compared to n = 107 (35%) who participated in GMV. 

Co-morbidities were extracted from the initial visit of the patient chart using diagnosis codes and included, but were not limited to, insulin resistance, hypertension, and obstructive sleep apnea. There were no differences between the IMV and GMV on diabetic status (22% and 28%, respectively); however, the total number of co-morbidities was slightly higher in the GMV group (*p* = 0.45) at the initial visit. 

**Figure 1:**
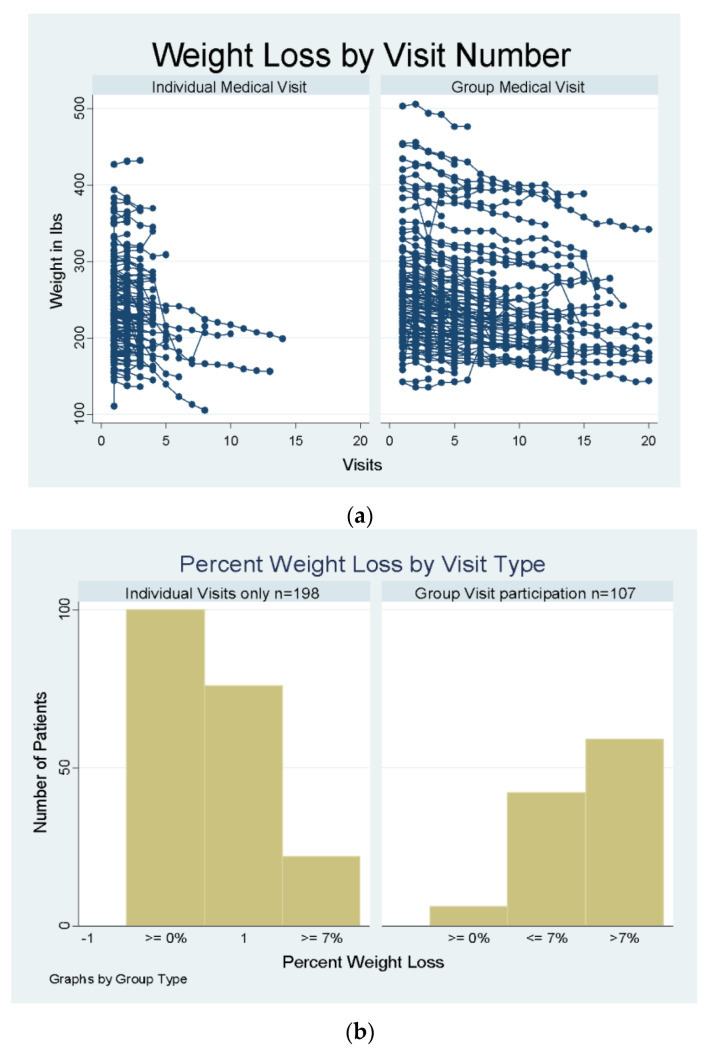
(**a**) Weight by Number of Visits in Each Group. (**b**) Percent Weight Loss by Visit Type.

Mixed-effects regression models indicated that patients in the IMV group lost an average of 3.99 lbs, whereas patients in the GMV settings lost an average of 11.63 lbs. This result remained statistically significant (*p* < 0.001) after adjusting for age, number of co-morbidities, start weight, and sex, as described in Table 1. These differences in weight loss between the IMV and GMV groups remained statistically significant when adjusting for the number of visits, indicating that the weight-loss differences were independent of the patient engagement effect. Most visits were bi-weekly, with the average number of visits attended being 5 and the range being from 1 to 43. Figure 1a shows a scatterplot of weight by number of visits for each group. 

Patients’ total weight loss (i.e., from initial weight to weight at their last visit) was categorized by whether they achieved 7% or more weight loss. Attaining a 7% weight loss is related to a reduction of diabetes risk or transitioning pre-diabetes to diabetes [23]. Among patients in the GMV, 55% of patients lost 7% or more weight compared to 11% of patients in the IMV category (*p* ≤ 0.001), as shown in Figure 1b. 

**Figure 2:**
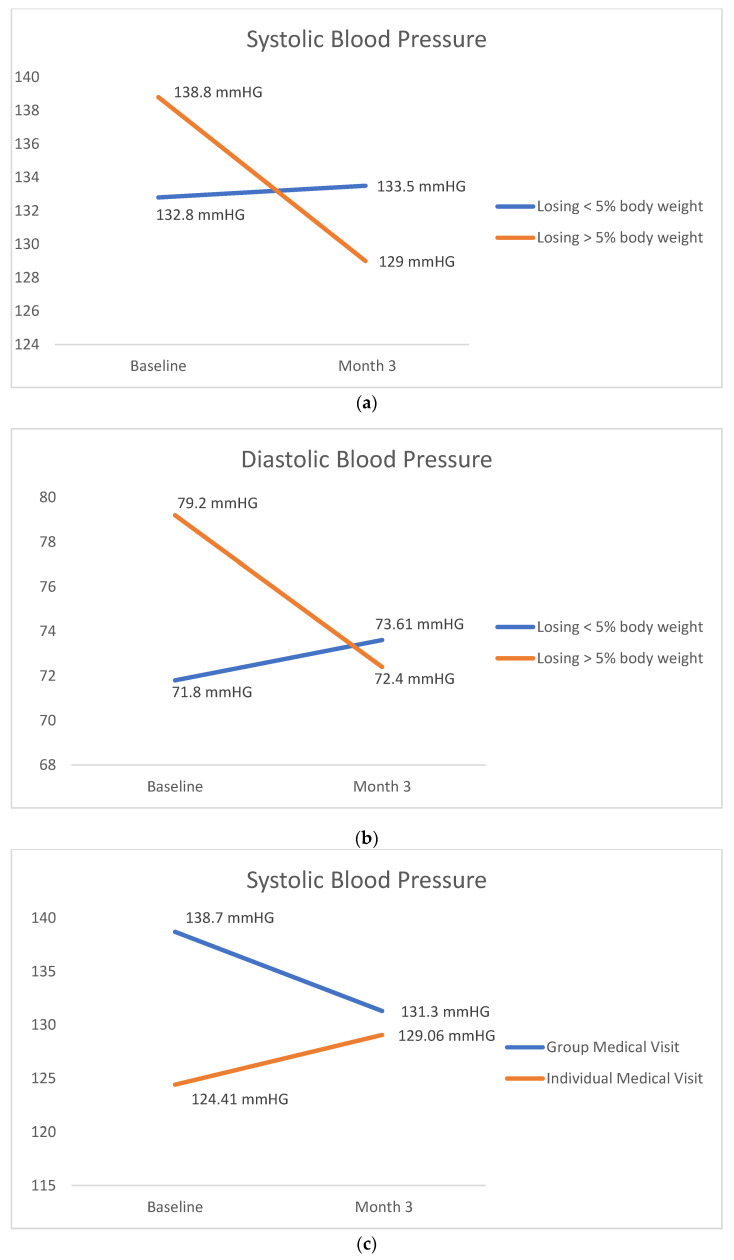

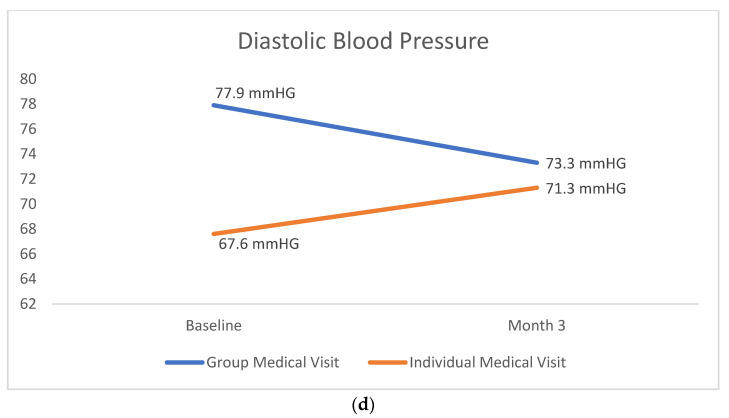
(**a**) Changes in Systolic Blood Pressure by % Weight Loss. (**b**) Changes in Blood Pressure by % Weight Loss. (**c**) Changes in Systolic Blood Pressure by Visit Type. (**d**) Changes in Blood Pressure by Visit Type.

Data for blood pressure were available from 81 patients. There were 22 subjects in the IMV and 59 subjects in the GMV. As shown in Figure 2a, the mean baseline systolic blood pressure for GMV and IMV were 138.7 mmHG, and 124.41 mmHg, respectively. The mean baseline diastolic blood pressure for GMV and IMV were 77.9 mmHg and 67.6 mmHG, respectively. The difference between baseline and three months systolic blood pressure in the GMV and IMV was −7.4 vs. 4.1 mm of Hg (*p* = 0.002), respectively. The difference for diastolic blood pressure between GMV and IMV, for baseline and three months, was −4.6 vs. 4.2 mm of Hg (*p* = 0.003), as shown in Figure 2d. Individuals were classified as either less than or greater than 5% weight loss. This level, 5% weight loss, is related to a reduction in metabolic markers such as blood pressure [24]. Those who lost >5% of their baseline weight had a higher reduction in overall blood pressure, as shown in Figure 2a. For systolic blood pressure, the difference between baseline and three months was −9.8 vs. 0.73 mm of Hg for subjects losing >5% weight compared to those losing ≤5% weight. For diastolic blood pressure, the difference between baseline and three months was −6.6 vs. 1.81 mm of Hg for subjects losing >5% weight compared to those losing ≤5% weight.

## 4. Discussion

This retrospective cohort study demonstrates that GMV may be an effective modality for lowering weight and blood pressure in a population of underserved patients. Patients attending GMV tended to stay in the program longer than those who attended IMV. Patients losing more than 5% of their body weight demonstrated greater reduction in blood pressure, with more patients in the GMV group achieving a >7% weight loss than those in the IMV group. These findings are in line with those described by Trento et al., who demonstrated a significant greater weight loss in diabetic patients in GMV (2.6 kg or 5.73 lbs) compared to IMV (0.9 kg or 1.98 lbs) [25]. The current study focused on all patients wanting to lose weight and not just those with diabetes or other underlying co-morbidities, thus demonstrating the generalizability of these findings to a wider patient population. 

The non-randomized nature of group category assignments in this real-world intervention is both a strength and a limitation of the study. The patients chose their preference of continuing with GMV or IMV after their initial visit and there were no restrictions on the number of sessions that they could attend, thus creating a risk of selection bias, but at the same time increasing the generalizability of the results in a real-world clinic setting. Patients attended as many visits as they felt comfortable, giving them greater control in customizing care to meet their needs. Analyses were adjusted for the number of visits to address this potential bias.

The novelty of this study lies in the setting in which it was conducted. The CWL program is embedded in a busy full-spectrum family medicine practice and a residency training program, serving a predominantly underserved racially and ethnically diverse population. As a result, the CWL program needed to adapt operations to fit within the workflow of the existing full-spectrum family medicine clinic, utilizing existing clinic staff, and adjusting the intervention to multiple parent clinic operational requirements. 

Although validation of these findings through appropriate designed prospective studies are needed, results from this study indicate that the medical group therapy model is a promising strategy for increasing the efficacy and efficiency of clinical weight-loss interventions and blood pressure control, especially among the high-volume resource-lacking practices that serve the underserved populations. 

## Figures and Tables

**Table 1:** Baseline characteristics in Individual Medical Visits (IMV) and Group Medical Visits (GMV).

	Individual Visits	Group Visits	Test of Differences
Total patients n (%)	198 (65%)	107 (35%)	
Age (mean SD)	44 (12.6) years	48 (11.3) years	*p* = 0.005
% Female	170 (86%)	91 (85%)	*p* ≥ 0.05
Race/Ethnicity			
White	78 (39%)	46 (43%)	*p* ≥ 0.05
Hispanic	94 (47%)	43 (40%)	
Black	11 (6%)	4 (4%)	
Other	15 (8%)	14 (13%)	
Start Weight (mean SD)	243.5 (54.5) lbs	260.3 (70.6) lbs	*p* = 0.02
# Comorbidities	6.6 (3)	7.3 (3.1)	*p* = 0.045
Prediabetes or Diabetes	44 (22%)	30 (28%)	*p* ≥ 0.05

## Data Availability

Not applicable.
